# Assessing Medical Students’, Residents’, and the Public's Perceptions of the Uses of Personal Digital Assistants

**DOI:** 10.3885/meo.2008.Res00258

**Published:** 2008-06-23

**Authors:** Pradip D. Patel, Ruth B. Greenberg, Karen Hughes Miller, Mary B. Carter, Craig H. Ziegler

**Affiliations:** * Department of Pediatrics, University of Louisville School of Medicine, Louisville KY, USA; † Office of Medical Education, University of Louisville School of Medicine, Louisville, KY, USA

**Keywords:** medical students, residents, public perception, handheld wireless devices, personal digital assistants (PDAs), standardized patients

## Abstract

Although medical schools are encouraging the use of personal digital assistants (PDAs), there have been few investigations of attitudes toward their use by students or residents and only one investigation of the public's attitude toward their use by physicians. In 2006, the University of Louisville School of Medicine surveyed 121 third- and fourth-year medical students, 53 residents, and 51 members of the non-medical public about their attitudes toward PDAs. Students were using either the Palm i705 or the Dell Axim X50v; residents were using devices they selected themselves (referred to in the study generically as PDAs). Three survey instruments were designed to investigate attitudes of (a) third- and fourth-year medical students on clinical rotations, (b) Internal Medicine and Pediatrics residents, and (c) volunteer members of the public found in the waiting rooms of three university practice clinics. Both residents and medical students found their devices useful, with more residents (46.8%) than students (16.2%) (p < 0.001) rating PDAs “very useful.” While students and residents generally agreed that PDAs improved the quality of their learning, residents’ responses were significantly higher (p < 0.05) than students’. Residents also responded more positively than students that PDAs made them more effective as clinicians. Although members of the public were generally supportive of PDA use, they appeared to have some misconceptions about how and why physicians were using them. The next phase of research will be to refine the research questions and survey instruments in collaboration with another medical school.

Physician use of personal digital assistants (PDAs) has increased dramatically,^1^,^2^ and many medical schools are now encouraging student use of the devices, in some cases financially supporting school-wide or clerkship-wide programs.^3^ However, the literature on medical student and resident use of these devices focuses almost entirely on small groups of users, for example, a single clerkship where medical students are using a PDA to record patient encounters.

One of the earliest studies on handheld device technology was conducted at the University of Cambridge with 13 students in a community-based experience. In 1999, Alderson and Oswald reported that the use of a handheld computer to record student contacts with patients produced more detailed descriptions of students’ clinical experiences and better record keeping.^4^ In 2000, Sullivan, Halbach, and Shu reported the results of a study involving students in a Family Medicine clerkship at New York Medical College.^5^ Their preliminary findings were that student use of a PDA to record patient contact information and preceptor evaluations was “an effective way to integrate current technology into the academic curriculum in measurable ways that enhance student learning and program evaluation” (p. 535).

At the graduate level, 13 Obstetrics and Gynecology residents at the Uniformed Services University of Health Sciences and the Walter Reed Army Medical Center piloted the use of a handheld device to track patient encounters.^6^ These investigators reported that using a PDA to collect resident patient encounter data had a positive impact on the education of the residents and that the handheld computers were “increasingly useful in the daily practice of clinical and academic medicine.” Each of these studies suggests that handheld technologies enhanced medical education; however, they focused on small groups of handheld device users and on one particular kind of application, the patient encounter log (p. 793).

An institution-wide pilot study was completed at Wayne State University School of Medicine on the utility of handheld wireless devices. While student utilization rather than student perception was the focus of the investigation, researchers did conclude that students were “coming to rely on handheld wireless devices to support their clinical decision making” (p. 8).^7^

Only one study thus far, Rudkin et al. in 2006, investigated patients’ attitudes toward physician use of PDAs in their presence.^2^ This study, conducted in an urban university hospital (but limited to emergency room encounters), found patients to be either neutral or positive in their attitudes toward PDA use by physicians in their presence.

The University of Louisville School of Medicine (U of L SOM) pilot study described in this paper was conducted during the first semester of the 2006–2007 academic year. It was the first study to examine perceptions of a wide group of medical students and residents from multiple disciplines and the first to examine perceptions of a diverse group of the non-medical public on physician use of PDAs in their presence. During this study, students were using either the Palm i705 or the Dell Axim X50v (without the telephone capabilities of a “smartphone”) all loaded with the same software. These models were issued to U of L SOM students at that time. Residents were using PDAs of their own choosing.

The purpose of the study was to expand the recent research on PDA use, specifically to (a) explore the attitudes of medical students and residents toward PDA use and (b) explore the attitudes of the non-medical public toward physician use of a PDA in their presence.

We explored the following hypotheses: (a) medical students and residents will share similar positive attitudes toward using a PDA to care for patients; and (b) members of the general public in a university practice waiting room will have positive attitudes toward physician use of a PDA in their presence.

## Methods

**Study Design -** Separate survey instruments were designed for each of the three research populations: (a) medical students; (b) residents; and (c) members of the non-medical public. Questions were designed to collect data on issues that emerged from a previous U of L SOM web-based student evaluation of the school's original PDA initiative,^8^ issues of practical interest to the Office of Medical Education, and issues identified in physician survey tools developed by Hamstra, Lamer, and Miller^9^ (with their permission) for use within the Indian Health Service. After obtaining Institutional Review Board (IRB) approval, surveys were administered in summer 2006 to a convenience sample of third- and fourth-year medical students, residents, and members of the non-medical public. Third- and fourth-year medical students from eight clinical rotations and residents from the departments of Pediatrics and Internal Medicine were invited to complete the survey instrument during regular class meetings or conferences. Members of the non-medical public were interviewed by standardized patients (SPs) trained as interviewers in the waiting rooms of three university practice clinics: Obstetrics and Gynecology, Pediatrics, and Family Medicine.

**Student and Resident Survey Items -** Student and resident surveys were designed to collect demographic data (i.e. age and gender), feedback on PDAs (including frequency of use), and personal reactions to using a PDA. Likert-scaled survey items asked students and residents to rate their PDA in terms of the following aspects of use: (a) clinical usefulness; (b) quality of learning, effectiveness as a clinician; (c) whether their use will increase over time; and (d) speculation about future hardware and software improvements. The five-point Likert scales were anchored with “Very Useful” and “Not Useful,” or “Strongly Disagree” and “Strongly Agree.” Items that asked about frequency of use were scaled as follows: 0 = Never, 1 = Almost never, 2 = A few times a month, 3 = A few times a week, 4 = Once a day, and 5 = At least twice a day. The mean ranking of the three “best features” and the three “biggest drawbacks” of PDA use identified most often by students and residents was tabulated. Finally, the surveys also asked about PDA use in the presence of patients and how the students and residents perceived patient reactions to PDA use.

**Public Survey Items -** The public survey contained demographic questions (i.e., age and gender), questions about the subject's use of and comfort level with computers, and a question about recognizing a PDA. Public perceptions of the tasks a physician could perform using a PDA were collected using “yes/no/don't know” options. The public survey also asked five Likert-scaled questions (anchored with “Strongly Disagree” and “Strongly Agree”) about attitudes toward technology and PDA use by physicians in their presence. At the end of the survey, non-medical participants were invited to comment about doctors and PDA use.

To neutralize the problem of variations in public literacy levels, SPs were trained to administer the public survey by reading each question out loud and writing the responses to the open-ended questions verbatim on the data collection sheets. SPs were used for this data collection because they were available, were easily trained, and already had the skills to present information consistently. Participating SPs completed IRB and Collaborative Institutional Training Initiative (CITI) training and additional training in the data collection process before meeting the public. SPs, perhaps because of their good social skills, proved to be highly effective interviewers. The directors of three university practice clinics, the Department of Pediatrics, the Department of Family and Geriatric Medicine, and the Department of Obstetrics and Gynecology, agreed to allow the SPs to interview people seated in their waiting rooms. Members of the public were invited to participate by an SP, and, if consent was given, the SP read the survey questions to the participant. The individual's responses were recorded on the instrument by the SP. In the case of children (ages 9 to 18), the parent or guardian was invited to sit in on the interview but was seated out of the child's range of sight so that his or her responses were unimpeded. As a gift rather than an incentive, at the end of the interview, SPs gave each participant a $5 fast food gift certificate.

SPSS (SPSS, 2003) version 14.0 was used to analyze the quantitative data. Likert-scaled data were analyzed using the Mann–Whitney *U* test. The Chi-square statistic or Fisher's exact test was used to analyze dichotomous data. Results were compared for medical students and residents using both inferential and descriptive statistics. Public data were analyzed separately because that instrument was markedly different, but similar statistical methods were applied. A comparison was made between responses of children (ages 9 to 18) and adults (19 and older), as well as between those of computer users and non-computer users. All *p*-values were two-tailed. Statistical significance was set by convention at *p*<0.05.

Finally, public responses to open-ended questions were compared using the constant comparison method first described by Glazer and Strauss in 1964 and refined by Dye, Schatz, Rosenberg, and Coleman in 2000.^10^ This qualitative analysis was conducted to clarify and refine results of the quantitative data.

## Results

**Students and Residents -** One hundred twenty-one third- and fourth-year medical students from a variety of clinical rotations and electives (Surgery 43%, Pediatrics 36%, Internal Medicine 7%, Family Medicine 3%, Neurology 3%, OBGYN 3%, Psychiatry 3%, Radiology 1%, and Pediatrics [Newborn Nursery] 1%) participated in this study, representing approximately 42% of the third- and fourth-year medical students at the institution. Of these, 44% were female and 82% were third-year students. The mean student age was 25.7 years (SD 2.3). Of the residents, 31 from the Department of Pediatrics and 22 from the Department of Internal Medicine participated, representing approximately 35% of the resident population in these two departments. Fifty-six percent were female, and the mean age was 28.7 years (SD 3.3).

A majority of students (75.5%) and residents (77.7%) reported that they currently use a PDA. However, since almost all of the self-reported “non-user” responses to survey items indicated they were familiar with the device, we included almost all student and resident data in the final analysis. Only the two non-users who offered no opinions were dropped from the analysis because of missing data.

**Figure 1:**
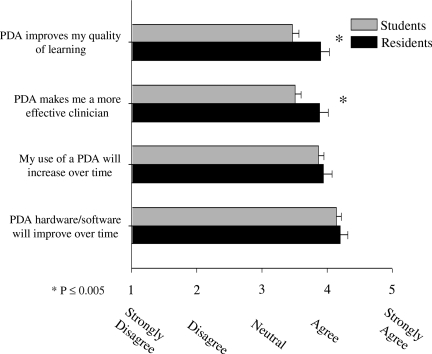
Mean PDA usage by medical student/residents†

Students differed from residents in their attitudes toward using a PDA and its effects on their learning and patient care. More residents rated the device “very useful clinically” (46.8%) than did medical students (16.2%, *p*<0.001). Significant differences between student and resident responses to items that asked if the device improved their quality of learning and made them more effective clinicians were also found (*p*<0.05, Figure 1). No differences were found for items regarding the projected increased use of the device over time or the hardware/software improving over time.

Statistically significant differences were also found in how often students and residents used their devices. Residents accessed drug information approximately once a day while students accessed drug information a few times a week (*p*<0.001). Similarly, residents used their devices more frequently than students to access information from a clinical reference application (*p*=0.05).

**Figure 2:**
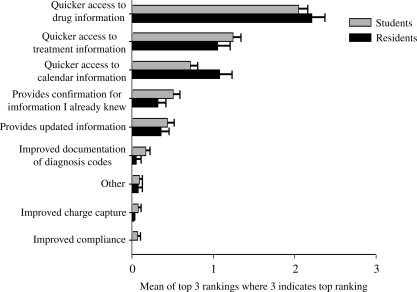
Best Features of PDA use by medical student/residents†

Students and residents agreed that the three best features of their devices were having (a) quicker access to drug information, (b) quicker access to treatment information and (c) access to their personal calendar. Students and residents both ranked having access to drug information highest. Residents ranked having access to treatment information similarly to access to their personal calendars, while students ranked having access to treatment information higher than having access to their personal calendars. (Figure 2)

**Figure 3:**
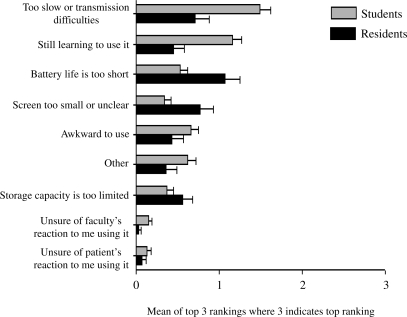
Biggest drawback of PDA use by medical student/residents†

In spite of the fact that students and residents were using a variety of PDA devices, they agreed that the major drawbacks of using the devices related to technological issues. Students selected “too slow” or “transmission difficulties” as the top drawback, while residents selected “battery life is too short” as the top drawback. Students rated learning to use the PDA and its awkwardness as the second and third drawbacks, respectively, while residents rated small screen size and speed or transmission difficulties second or third, respectively. (Figure 3)

**Figure 4:**
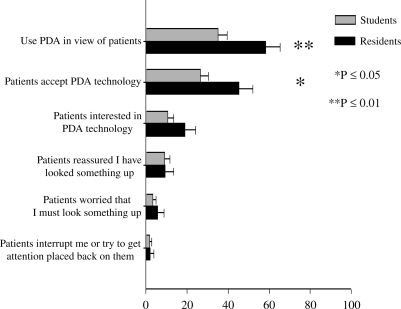
Percentage Yes to inferred patient's perception of PDA use by medical students/residents†

Because of differences in clinical opportunities, students used their device in the presence of patients less often than residents; 58.3% of the residents reported using their device in front of patients compared to 35.1% of students (*p*=0.009, Figure 4). Almost 73% of the residents and 68% of the students agreed or strongly agreed that, over time, their patients would become more comfortable seeing clinicians use a PDA (residents, mean = 3.90; students, mean = 3.71, *p*=0.178). When asked to infer patient attitudes toward PDA use by a clinician, only 26.4% of students believed that their patients “accept PDA technology” as compared to 45.3% of the residents (*p*=0.021). Residents and students held similar views of their patients’ level of interest in PDA technology, their patients’ reaction toward seeing a physician use a device to look something up, and their patients’ desire to get a physician's attention focused back on them when he or she was using a device.

**Members of the Public -** Fifty-one members of the public, representing 100% of those approached, agreed to be interviewed for this study. The mean age of the 51 non-medical participants was 28.8 years (min 9, max 67, SD 13.0). Nine participants were age 18 and under; 74% were female. Forty-four (86%) used a computer at home, school, or work. Only one (2%) reported using a PDA. Of the 51 members of the public who participated in this study, 84% could identify the device shown to them, a Dell Axim X50v. Thirty-seven percent referred to the device as a “Palm Pilot”. 31% as a “PDA”, 8% as a “wireless notebook or notepad”, and one each as a “notebook,” “organizer,” “hand computer,” and “iPod.” Adults were more likely than children to identify the PDA (adults, 90%; children 56%; *p*=0.026, Fisher's exact test), and 39% of the non-medical public had seen a physician use a PDA. Participant computer usage did *not* correlate with ability to identify the PDA by some name.

When asked about the tasks their physician could perform on a PDA, most non-medical participants knew that a physician could check a calendar and schedule (98%), write a reminder note (96%), read or send email (74%), and look up information about drugs (63%) and how to treat an illness (57%). However, less than half of the non-medical participants knew that a physician could use a PDA to check drug–drug interactions (49%), look up information about a patient (47%), look up information about a patient's medical insurance (28%), or use it as a cell phone (18%). All adult participants (100%) were aware that physicians could use the PDA to write a reminder note in comparison to 78% of children (*p*=0.029, Fisher's exact test), while adults were almost twice as likely as children to be aware that doctors could use a PDA to read or send email (adults 83%; children, 44%; *p*=0.027, Fisher's exact test). There were no differences in perceptions of PDA function between computer users and non-users.

**Figure 5:**
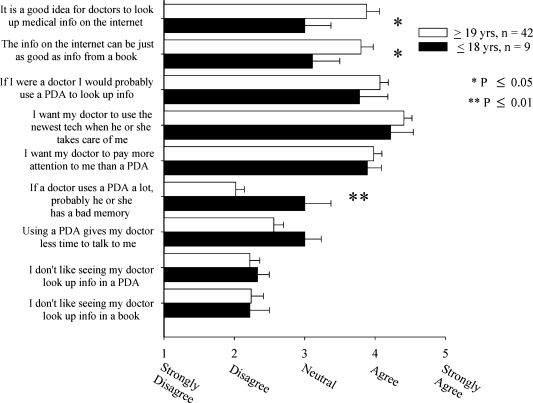
Public perception of technology by age group†

In general, members of the non-medical public were supportive of technology and of physician PDA use. When differences between children and adults were analyzed, results for two positive statements (“it's a good idea for doctors to look up information on the internet” (*p*=0.025) and “information on the internet can be just as good as information from a book” (*p*=0.05)) were significantly higher for adults. In contrast, children were more likely to rate the statement, “if a doctor uses a PDA a lot, he or she probably has a bad memory” higher than adults (*p*=0.003). In general, children were more likely to give neutral replies, but no differences were found between computer users and non-users in the non-medical population as a whole in regard to physician PDA use. (Figure 5)

After completing the survey, members of the non-medical public were asked if they had additional comments. Nineteen of the 51 people interviewed made comments. When their replies were analyzed using an updated version of the constant comparison technique, four major concepts emerged. Examples are presented in Table 1. The majority of comments focused on the PDA's function in *supporting physicians* and used words such as “organized,” “helpful,” and “resourceful.” The second most frequent concept was *benefit to physicians and patients*. Words and phrases such as “faster” and “benefits all” were used. Three people expressed caveats such as “use the device on breaks rather than in front of patients” and not “abusing the privilege by playing games.” One respondent voiced a concern about lost PDAs and personal information. Only two comments provided no usable data.

**Table 1:** Qualitative Data, Non Medical Public

Major Concept	Descriptors	Frequency*
Supports physicians	Helps doctors stay organized; helps them know what they are talking about; makes doctors more resourceful.	8
Benefits both physician and patient	Makes things faster for everybody; available to give patients best care; it benefits all; taking good care of me because they are using the best technology.	5
Caveats	They should use it on breaks and not in front of patients; they should not abuse the privilege (by playing games etc.); could be helpful but if the wrong doctor gets it he might put his personal information on it….	3
Personal experience	It's been helpful in my medical situation.	1

*19 non-medical participants volunteered open ended comments

## Discussion

This investigation confirms previous findings with individual clerkships and residencies that students and residents perceive their PDA as useful. However, it also explores student, resident, and the non-medical public's attitudes toward PDA use. Our findings indicate that while students and residents have a generally positive view of PDA use, residents have a more patient-centered view and believe more strongly that their use of a PDA has a positive impact on their ability to care for patients.

The majority of students and residents surveyed use their PDA, although residents reported using their PDA more often than students (daily vs. a few times each week). Similarly, although residents and students agree that the best features of a PDA relate to patient care (i.e., quicker access to drug and treatment information), residents were more likely than medical students to agree that using a PDA improved the quality of their learning and made them more effective clinicians. Differences between student and resident perceptions may be related to the actual time spent providing patient care. Since residents spend more hours providing direct patient care, their PDA use is greater, which may influence their general attitudes toward using a PDA. This possibility suggests our first question for future research: If students had a better understanding of the value of PDAs in their studies and in patient care, would their attitudes be even more positive?

It is also possible that perceptions about the value of PDA use may be related to familiarity with the technology and ease of PDA use. For example, residents rated “battery life is too short” as the biggest drawback of PDA use, a technological problem that is easily addressed. In contrast, students rated “still learning to use it” as the biggest drawback and “awkward to use” as the second major drawback, two drawbacks that may not be addressed as easily. Since it is possible that some students were hampered by their lack of skill in using their PDAs, a second possible new research question emerges: Would additional PDA training to provide students with the skills that residents appear to possess have a positive impact on students’ perception of PDA utility?

Members of the non-medical public, regardless of their familiarity with technology, were generally supportive of physician use of PDAs. This finding is similar to that of Rudkin et al.^2^ In addition, members of the public generally recognized that PDAs are useful to physicians and were not overly concerned that physician use of a PDA would take away time from their personal interactions with their physicians.

However, quantitative data from yes/no/don't know questions and qualitative data from the open-ended questions on the public survey instrument suggest that both adults and children have some misconceptions about what physicians actually *do* with their PDAs. Comments suggest that they believe the information on the PDA is static; they do not realize that a physician using a PDA may be calculating a drug dosage specifically for them or determining how a drug he/she is planning to prescribe will interact with drugs that the patient is already taking. In other words, the non-medical public may not differentiate between the open source, non-interactive information they can locate on the Internet for themselves and the proprietary, interactive software that supports clinical decision making. Although they express a fairly high level of confidence in web-based information, they do not realize how the highly specialized PDA accessed information is being used by physicians. This finding suggests our third question for possible future research: Would having additional knowledge about the proprietary, interactive software available on a physician's PDA have a positive impact on the non-medical public's perception of PDA use?

Four limitations of this study were the sample size, the single institution design, one vaguely worded question that asked students and residents if they were PDA “users,” and the exploratory nature of the study. These limitations will be addressed in a future study planned for 2008 in which we will expand the number of institutions and subjects, refine the three instruments, and add new questions that address our new research questions. Increasing sample sizes will increase statistical power. In addition, since this is the first investigation to explore medical student and resident attitudes and perceptions of PDA use, our future investigation will explore the factors that influence student, resident, and faculty use of PDAs. For example, does using a PDA in medical school affect PDA use as a resident?

An additional possible weakness of the study was that multiple testing was performed in an exploratory fashion on the different survey questions, possibly inflating the Type 1 error rate. However, because the statistically significant mean increase of residents over students on the items asking if the “PDA improved the quality of their learning” and “PDAs make students more effective clinicians” are logical findings, Type I errors probably did not occur. For the general public population, because of the small sample of children interviewed, it is plausible their perceptions were not representative of the population of children as a whole; hence, it is somewhat more probable that the significant differences found in this study between children and adults are Type 1 errors, and some caution should be taken in accepting the findings. In future studies, larger populations will allow us to more convincingly distinguish differences between children and adults.

A positive outcome of this study was the successful use of SPs to collect data from the non-medical public in university practice waiting rooms. A self-selected group of SPs completed Collaborative CITI training on human subjects research and, perhaps because of their high level of social skills, were found to be very effective interviewers. The varying levels of public literacy were no longer an issue since SPs were able to read the questionnaires to each subject and capture all open-ended comments correctly. We believe this process improved the face validity of the non-medical public data.

As medical students, residents, and medical faculty learn to rely on handheld wireless devices for even more applications, we must continue to explore not only these applications but also attitudes, perceptions and understanding. As more members of the non-medical public see PDAs used in their presence, we must also explore their attitudes and perceptions. These data could be used to inform our decisions about how PDAs can be used to enhance teaching, learning, and patient care without diminishing personal interactions.

## Footnotes

†Error Bars Represent one standard error of the mean (SEM)

†Error bars represent one standard error of the mean (SEM)

†Error bars represent one standard error of the mean (SEM).

†Error bars represent one standard error of the mean (SEM).

†Error bars represent one standard error.

## References

[1] Garritty C, El Emam K (2006). Who's using PDAs? Estimates of PDA use by health care providers: a systematic review of surveys. J Med Internet Res.

[2] Rudkin SE, Langdorf MI, Macias D, Oman JA, Kazzi AA (2006). Personal digital assistants change management more often than paper texts and foster patient confidence. Eur J Emerg Med.

[3] Wake Forest University Baptist Medical Center. Wake Forest medical center using palm OS PDAs to train tomorrow's doctors. pdaMD.com.

[4] Alderson T, Oswald NT (1999). Clinical experience of medical students in primary care: use of an electronic log in monitoring experience and in guiding education in the Cambridge community based clinical course. Med Educ.

[5] Sullivan L, Halbach JL, Shu T (2001). Using personal digital assistants in a family medicine clerkship. Acad Med.

[6] Malan TK, Haffner WH, Armstrong AY, Satin AJ (2000). Hand held computer operating system program for collection of resident experience data. Obstet Gynecol.

[7] Jackson M, Ganger A, Bridge P, Ginsburg K (2005). Wireless hand held computers in the undergraduate medical curriculum. Med Ed Online.

[8] Greenberg R (2004). Use of the personal digital assistant (PDA) in medical education. Med Educ.

[9] Hamstra S, Lamer C, Miller C (2002). Personal digital assistants in the Indian Health Service. The IHS Provider.

[10] Dye JF, Schatz IM, Rosenberg BA, Coleman ST (2000). Constant Comparison Method: A kaleidoscope of data. The Qualitative Report.

